# Xuebijing Injection Alleviates Pam3CSK4-Induced Inflammatory Response and Protects Mice From Sepsis Caused by Methicillin-Resistant *Staphylococcus aureus*

**DOI:** 10.3389/fphar.2020.00104

**Published:** 2020-02-21

**Authors:** Tiantian Li, Yiming Qian, Zhulei Miao, Peiyong Zheng, Ting Shi, Xinru Jiang, Lingyun Pan, Fenghua Qian, Guizhen Yang, Huazhang An, Yuejuan Zheng

**Affiliations:** ^1^ Department of Immunology and Microbiology, School of Basic Medical Sciences, Shanghai University of Traditional Chinese Medicine, Shanghai, China; ^2^ Department of Emergency, Yueyang Hospital of Integrated Chinese and Western Medicine affiliated to Shanghai University of Traditional Chinese Medicine, Shanghai, China; ^3^ Institute of Digestive Diseases, Longhua Hospital affiliated to Shanghai University of Traditional Chinese Medicine, Shanghai, China; ^4^ Experiment Center for Science and Technology, Shanghai University of Traditional Chinese Medicine, Shanghai, China; ^5^ Clinical Cancer Institute, Center of Translational Medicine, Second Military Medical University, Shanghai, China

**Keywords:** sepsis, methicillin-resistant *Staphylococcus aureus*, Pam3CSK4, inflammatory cytokines, macrophages, Xuebijing injection

## Abstract

A leading cause of death worldwide is sepsis that develops as a dysregulated immune response to infection. Serious infection caused by methicillin-resistant Staphylococcus aureus (MRSA) increases the difficulty of treatment in septic patients. Host-directed therapy (HDT) is an emerging approach to bacterial infections. Xuebijing injection (XBJ), a commercialized injectable prescription from traditional Chinese medicine, has been used as adjuvant therapy for sepsis with a history of 15 years. Whether it plays a protective role in severe infection caused by antibiotic-resistant bacteria is still unknown. In this study, XBJ significantly improved the survival of MRSA-induced sepsis mice. In MRSA-infected mouse model, XBJ down-regulated the expression of inflammatory cytokines interleukin (IL)-6, tumor necrosis factor (TNF)-α, MCP-1, MIP-2, and IL-10 in sera. Besides that, it decreased the bacterial load in spleens, livers, and alleviated tissue damage of lung, liver, and kidney. The combination of XBJ with vancomycin or dexamethasone exhibited a better down-regulatory role of the inflammatory response. Then, the protective mechanism of XBJ was further investigated. XBJ inhibited heat-killed MRSA-induced IL-6 and TNF-α production in mouse macrophages. XBJ also decreased Pam3CSK4 (a synthetic tripalmitoylated lipopeptide mimicking bacterial lipoproteins)-stimulated expression of IL-6, TNF-α, IL-1β, IL-12, etc. in mouse macrophages. Furthermore, XBJ down-regulated the activation of NF-κB, MAPK, and PI3K/Akt pathways in Pam3CSK4-stimulated mouse macrophages. In conclusion, our findings demonstrated that XBJ played a protective role in MRSA-challenged mice and down-regulated the inflammatory response and the activation of signaling pathways initiated by Pam3CSK4. It enlarged the clinical application of XBJ in the treatment of severe bacterial infection, e.g. caused by MRSA.

## Introduction

Sepsis is a life-threatening multi-organ dysfunction caused by a dysregulated immune response to infection (Fernando et al., 2018). Despite the remarkable progress achieved in clinical management, sepsis is still a burdensome international public health problem due to high morbidity, substantial mortality, and escalating costs associated with the increased complexity of care. It was estimated that 30 million cases of sepsis each year led to more than 8 million deaths. Furthermore, mortality for severe sepsis is 15–30% in high-income countries, while it is 50% or higher in low-and middle-income countries (Dugani et al., 2017). Rapid control of pathogens is given priority in the treatment of sepsis. First-time administration of broad-spectrum antibiotics has greatly improved the outcome of sepsis; however, long-term high-dose use of antibiotics will increase screening pressure of antibiotic-resistance of bacteria.

As a common Gram-positive (G^+^) bacteria, methicillin-resistant *Staphylococcus aureus* (MRSA) is resistant to multiple antibiotics in health care facilities and community settings. A review of 15 clinical investigations showed 13–74% of worldwide *S. aureus* infections were caused by MRSA (Kock et al., 2010). It was reported by China Antimicrobial Resistance Surveillance System (CARSS) that the annual detection rate of MRSA out of *S. aureus* in China was more than 32% during the past 5 years. Though the incidence of invasive MRSA infection has decreased, it still exhibits high morbidity and mortality (Hassoun et al., 2017). It was estimated that there would be 218,000 cases of MRSA infection per year if without any intervention. This can result in an annual economic burden of approximately $3.3 billion in United States (Gidengil et al., 2015). Thus, new therapies for MRSA infection are eagerly needed.

The severity of damage caused by infection depends on the load and virulence of the organism in addition to the host response that is directed against conserved molecular patterns that can be found in pathogenic microorganisms called pathogen-associated molecular patterns (PAMPs). The classical PAMPs of *S. aureus* such as peptidoglycan (PGN), lipoteichoic acid, and lipoproteins, are believed to activate innate immune cells, etc. They detect invading microorganisms *via* pattern recognition receptors (PRRs). Toll-like receptors (TLRs) are a specific family of PRRs for host cells to recognize different PAMPs. TLR2-based signaling in response to *S. aureus* infection plays a critical role in the initiation of an effective innate response (Chen and Alonzo, 2019). During *S. aureus* infection, PAMPs recognized by TLR2 trigger the production of inflammatory mediators such as interleukin (IL)-6, tumor necrosis factor (TNF)-α, IL-1β, nitric oxide (NO), chemokines, interferons, and IL-10 through the activation of TLR2-mediated nuclear factor κB (NF-κB), mitogen-activated protein kinase (MAPK), and phosphatidylinositol 3 kinase (PI3K)/Akt pathways (Takeuchi and Akira, 2010). Systemic inflammatory response syndrome, an initial hyper-inflammatory phase, is a prevalent feature of patients with sepsis (Simpson, 2018). This stage is marked by a burst in the production of inflammatory mediators due to an autoamplified activating cascade known as “cytokine storm”. It leads to the impaired contractility of blood vessels, increased microvascular permeability, reduced cardiac index, and increased coagulation, all of which result in tissue damage and even multiple organ dysfunction (Angus and van der Poll, 2013; Gotts and Matthay, 2016; Chousterman et al., 2017). Besides antibiotics, early therapeutic interventions (e.g. fluid resuscitation, administration vasopressors, etc.) elevated the survival rate of septic patients (Rhodes et al., 2017). It was reported that anti-inflammatory treatment strategies are hopeful in the early stage of sepsis (Venet and Monneret, 2018).

Host-directed therapy (HDT) is an emerging and viable adjuvant approach in the field of anti-infective therapy. HDT targets host immune system to enhance protective immune response, reduce exacerbated inflammation, and balance immune reactivity. It could alleviate immunopathology and benefit the outcome of treatment while bypassing the problem of antibiotic resistance (Zumla et al., 2016; Kaufmann et al., 2018). A few novel drug candidates with the strategy of HDT have been applied for the treatment of sepsis. For example, scutellarin from *Erigeron breviscapus* (Vant.) improves the survival of mice with bacterial sepsis by reducing serum IL-1β levels and attenuating the infiltration of inflammatory cells (Liu Y. et al., 2018). Curcumin, which is obtained from the rhizome of *Curcuma longa*, alleviates sepsis-induced acute organ injury by preventing inflammation and enhancing the suppressive function of T regulatory cells (Chen et al., 2018). Our previous studies showed that micheliolide, isolated from *Michelia compressa* (Magnoliaceae), can help maintain immune equilibrium and decrease PGN, *S. aureus*, and MRSA-triggered inflammatory response (Jiang et al., 2017).

Currently, natural products have proven to be outstanding sources of medicines. It offers unique and specialized chemical characteristics that engage with biological targets more efficiently (Wright, 2019). It is inspiring to look for immunoregulatory drug candidates from the abundant sources of natural products. A mixed composition of different natural products is usually used in traditional Chinese medicine (TCM) to treat different kinds of diseases. The most important characteristic of TCM is to maintain immune balance by the theory of Yin (negative regulation) and Yang (positive regulation) (Cao, 2008). Xuebijing injection (XBJ), an injectable prescription from TCM, is prepared from a combination of Honghua (*Carthami tinctorii* L.), Chishao (*Paeonia lactiflora* Pall.), Chuanxiong (*Ligusticum chuanxiong* Hort.), Danggui [*Angelica sinensis* (Oliv.) Diels], and Danshen (*Salvia miltiorrhizae* Bge.). This injectable five-herb preparation is available as a sterile, non-pyrogenic solution for intravenous administration. It has been approved by China Food and Drug Administration (China FDA; Beijing, China) in 2004 to treat systemic inflammatory response syndrome, sepsis, and multiple organ dysfunction in clinical guidelines (Yin and Li, 2014; Cheng et al., 2016). It is extensively used as an assistant therapy in the conventional management of sepsis in China. The latest randomized controlled trial showed that a standard therapy (such as antibiotics) combined with XBJ significantly improved the primary endpoint of pneumonia severity score, duration of mechanical ventilation and stay in intensive care units, and even mortality in critically III patients with severe community-acquired pneumonia (Song et al., 2019). Besides, XBJ also has therapeutic effects against heatstroke, severe burns, and paraquat poisoning (Dai et al., 2017; Gong P. et al., 2015; Xu et al., 2015). Previous studies have shown that XBJ may protect endothelial cells, improve microcirculation and coagulopathy, attenuate inflammatory reaction and regulate anti-oxidative stress, etc. (Xu et al., 2015; Wang et al., 2016; Chen et al., 2017; Zuo et al., 2018). Although the beneficial effects of XBJ have been widely investigated, the specific molecular mechanisms of XBJ are still obscure. Whether XBJ plays a protective role during the infection caused by drug-resistant bacteria (e.g. MRSA) has not been explored. Vancomycin (VAN), a glycopeptide antibiotic, is widely used in severe infection caused by G^+^ bacteria, especially drug-resistant G^+^ bacterial infection, including MRSA (Holland et al., 2014). Glucocorticoid dexamethasone (DXM) is regularly used in cases judged as a high initial inflammatory response in patients with sepsis (Annane et al., 2015). The combinational use of XBJ and VAN or DXM in MRSA-induced sepsis mouse model was explored in this study.

## Material and Methods

### Materials and Reagents

XBJ was purchased from Tianjin Chase Sun Pharmaceutical Co., LTD (Tianjin, China) with the batch number of 1811271 and stored at 4°C in the dark. Standards including nine analysis compounds were purchased from the Shanghai Tauto Biotech Co., Ltd (Shanghai, China).

### Chromatographic and Mass Spectrometry Conditions

The ultra-performance liquid chromatography-mass spectrometry (UPLC-MS/MS) analysis of XBJ was performed on a Waters Acquity Ultra Performance Liquid Chromatography system (Waters Corporation, Milford, MA, USA) coupled with a QTRAP 6500^+^ triple quadrupole mass spectrometer (Applied Biosystems/MDS Sciex, CA, USA) equipped with a turbo ion spray source. The chromatographic separation was achieved on a Waters Acquity UPLC BEH C18 (2.1 mm × 100 mm, 1.7 μm) at 35°C. The mobile phase consisted of A (water containing 0.1% formic acid) and B (acetonitrile). The gradient elution was conducted as follows: 0–1 min at 5% B, 1–8 min at 5–60% B, 8–10 min at 60–90% B, 10–12 min at 5% B. The flow rate was set at 0.3 ml/min and the injected volume was 5 µl.

The quantification was performed using multiple reaction monitoring (MRM). The data for optimal Q1 and Q3 mass, declustering potential (DP), collision energy (CE), and collision cell exit potential (CXP) are shown in **Supplementary Table S1**. The detection settings for mass spectrometer were as follows: Ion spray voltage, 5500 V (+) and 4500 (−); Entrance potential, 10 V (+); Curtain gas, 241.3 kPa; CAD, 48.3 kPa; Source temperature, 500°C Nebulizer gas (Gas 1), 413.7 kPa; Heater gas (gas 2), 413.7 kPa.

### Mice

C57/BL6J mice (6 weeks old, 16–18 g) were purchased from the Joint Ventures Sipper BK Experimental Animal Co. (Shanghai, China) and housed in a pathogen-free facility with free access to both water and food, kept in a 12 h light/dark cycle with controlled humidity (60–80%) and temperature (22 ± 1°C). All mice were adaptively fed at least 1 week before the experiment. All the experimental protocols were carried out in accordance with National Institute of Health Guide for the Care and Use of Laboratory Animals and approved by Shanghai Public Health Clinical Center Laboratory Animal Welfare and ethics committee with the number of 2019-A006-01.

### Cell Culture

Raw264.7 macrophage cell line was obtained from ATCC (Manassas, VA, USA) and cultured in Dulbecco's modified Eagle medium (DMEM) supplemented with 10% fetal bovine serum (FBS). Thioglycolate-elicited mouse primary peritoneal macrophages were prepared from female C57BL/6J mice (6–8 weeks of age) as described previously (Jiang et al., 2017). Adherent cells were used as mouse primary peritoneal macrophages after 2 h. Immortalized bone marrow-derived macrophages (iBMDMs) were maintained in RPMI-1640 medium supplemented with 10% FBS and 20 ng/ml macrophage colony-stimulating factor (PeproTech, Rocky Hill, NJ, USA) (Liu Z. et al., 2018). All cells were cultured at 37°C in a humidified incubator containing 5% CO2.

### Bacterial Strains, Growth Condition, and Preparation of Heat-Killed MRSA

MRSA strain HS488 was isolated from the bloodstream of a patient with severe sepsis, identified and numbered by the Institute of Antibiotics, Huashan Hospital affiliated to Fudan University, which was used in our *in vitro* and *in vivo* experiments. MRSA was incubated in Luria-Bertani (1% Tryptone, 0.5% Yeast Extract, 1% NaCl) broth at 37°C for 12 h with shaking at 250 rpm. Then, the growth medium containing bacteria were collected and the optical density of each well was determined at 600 nm using a Synergy 2 Microplate Reader (Bio-Tek, Vermont, USA) to calculate the concentration.

Suspension of heat-killed MRSA (HK-MRSA) was obtained through heating at 90°C for 30 min and then washed twice with phosphate-buffered saline (PBS). The viability of HK-MRSA was checked by culturing on agar plates overnight.

### MRSA-Induced Sepsis Mouse Model

Seven-week old C57/BL6J male mice were randomly divided into seven groups in the survival test: MRSA (n=13), MRSA+XBJ (5 ml/kg) (n=13), MRSA+XBJ (10 ml/kg) (n=13), MRSA+DXM (7 mg/kg) (n=13), MRSA+DXM (7 mg/kg) +XBJ (5 ml/kg) (n=13), MRSA+VAN (110 mg/kg) (n=10), and MRSA+VAN (110 mg/kg)+XBJ (5 ml/kg) (n=10). All the mice were challenged by intraperitoneal injection of a lethal dose of MRSA [7 × 10^8^ colony-forming unit (CFU)/mouse] and treated simultaneously with XBJ, VAN, and DXM as indicated. Survival status was recorded in the following 7 days.

### Determination of Bacterial Load of Spleens and Livers

At 12 h after MRSA challenge (2 × 10^8^ CFU/mouse) with the administration of XBJ, VAN, and DXM as indicated, spleens and livers of mice in each group were harvested (n=4/group), weighed, and homogenized in 1 ml of sterile 0.9% NaCl. After serial 10-fold dilutions in sterile 0.9% NaCl, 100 µl of each organ homogenates of three proper serial dilutions was aseptically plated on Luria-Bertani agar plates. CFU was counted and bacterial load was expressed as Log_10_ (CFU/g) per organ after 18 h of incubation at 37°C.

### Measurement of Levels of Inflammatory Mediators

Concentrations of IL-6, TNF-α, IL-12p70, IL-10, MCP-1, and MIP-2 were measured using ELISA kits according to the manufacturer's instructions (R&D Systems, MN, USA). Nitrite accumulation in supernatants was determined by Griess reagent [equal volume of 0.4% (w/v) 4-aminobenzenesulfonic acid in 20% (v/v) hydrochloric acid and 0.2% (w/v) N-(1-Naphthyl)-ethylenediamine dihydrochloride in water] and incubated in the dark at room temperature for 15 min. The absorbance at 540 nm was measured using a Synergy 2 Microplate Reader (Bio-Tek, Vermont, USA).

### Histopathology

Female C57BL/6 J mice were injected intraperitoneally with MRSA (4 × 10^8^ CFU/mouse), XBJ, VAN, and DXM as indicated. After 12 h, all mice were killed and lungs, livers, and kidneys were removed, washed with PBS, and fixed with 4% formaldehyde and paraffin-embedded. The tissues were sliced and stained with hematoxylin and eosin (H&E). The histopathological changes were observed using an Axio Imager M2 microscope (Carl Zeiss MicroImaging, Goettingen, Germany).

### Quantitative RT-PCR

Total RNA was isolated from cells using TRIzol reagent (Invitrogen, Carlsbad, CA) according to the manufacturer's instructions. Complementary DNA was synthesized from 0.5 μg total RNA by Prime Script RT reagent Kit (Takara, Dalian, China). Quantitative real-time PCR (qRT-PCR) analysis was performed with SYBR RT-PCR Kit (Takara, Dalian, China) and LightCycler (Roche Diagnostics, Indianapolis, IN) as described previously (Jiang et al., 2017). The expression levels of the mRNA were shown as fold change and normalized by the level of β-actin in each sample.

### Western Blot Analysis

Mouse primary peritoneal macrophages were stimulated by Pam3CSK4 and XBJ for various periods. Cell extracts were detected by Western blot as described (Jiang et al., 2017). All the examined antibodies were obtained from Cell Signaling Technology (Danvers, MA, USA).

### Statistical Analysis

All the data were presented as means ± standard deviation (SD). Comparisons between two groups were done using Student's *t* test analysis. Survival analysis was done using the log-rank test. Statistical significance was determined at p < 0.05, p < 0.01, and p < 0.001. The survival curve and plots of bacterial load were drawn by GraphPad Prism 8.0.

## Results

### UPLC-MS/MS Analysis of XBJ

**Figure 1:**
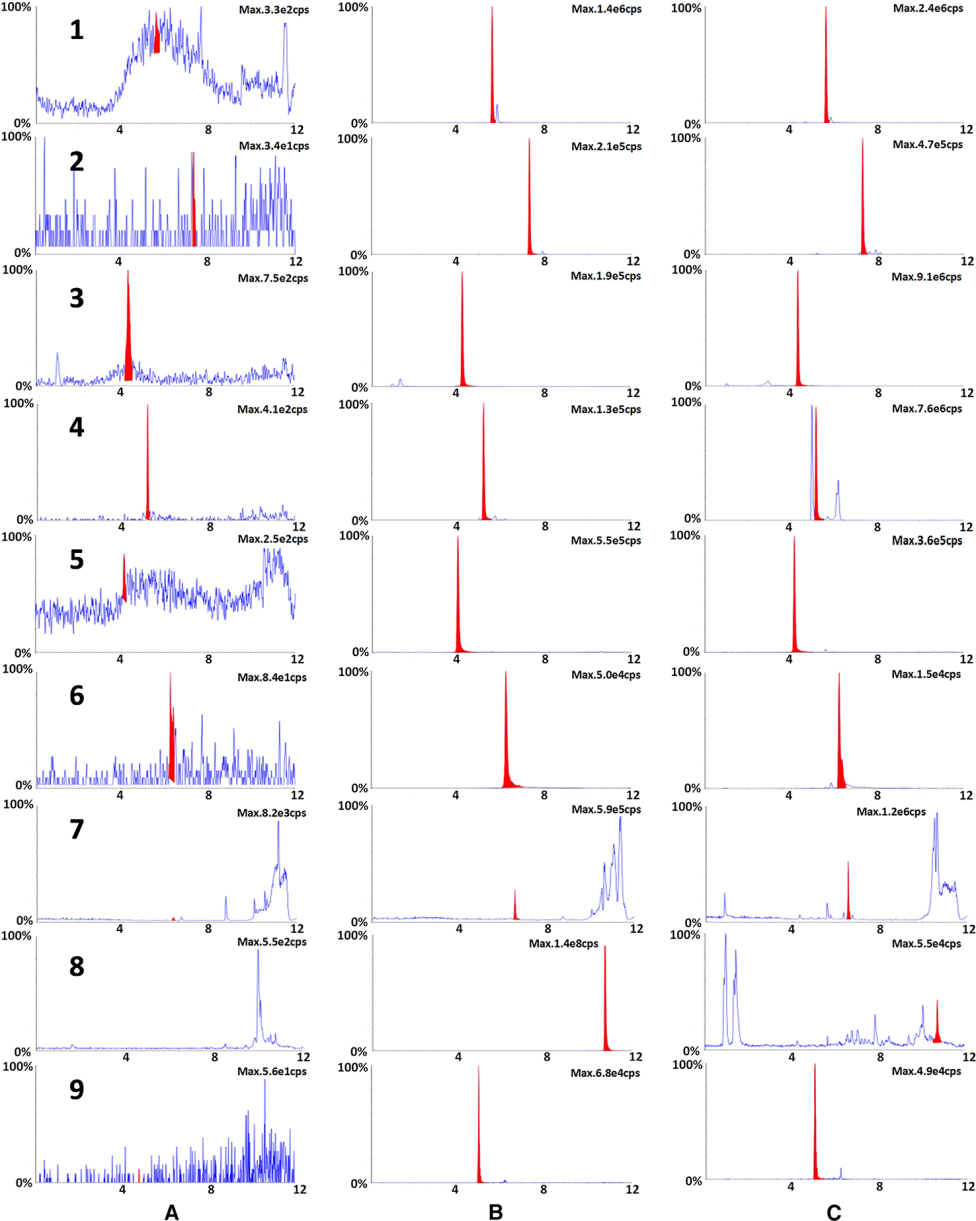
The typical chromatograms of blank **(A)**, a standard mixture of nine markers **(B)** and XBJ sample **(C)**. Nine representative bioactive ingredients were identified in XBJ using UPLC-MS/MS analysis: 1. ferulic acid, 2. benzoylpaeoniflorin, 3. HSYA, 4. PAE, 5. protocatechuic aldehyde, 6. salvianolic acid B, 7. senkyunolide I, 8. tanshinone IIA, 9. alibiflorin.

According to Chinese Pharmacopoeia and previous reports (Huang et al., 2011; Sun et al., 2017), nine representative bioactive compounds were identified in XBJ using UPLC-MS/MS analysis (**Figure 1**). These compounds included hydroxysafflor yellow A (HSYA), paeoniflorin (PAE), ferulic acid, tanshinone IIA, salvianolic acid B, benzoylpaeoniflorin, alibiflorin, senkyunolide I, and protocatechuic aldehyde. Their contents were 1373.33 ± 76.38 µg/ml, 2666.67 ± 245.42 µg/ml, 1.32 ± 0.84 µg/ml, 0.06 ± 0.02 µg/ml, 2.02 ± 0.84 µg/ml, 1.82 ± 1.08 µg/ml, 631.38 ± 361.07 µg/ml, 16 ± 11.6 µg/ml, and 0.38 ± 0.25 µg/ml respectively by three duplicates of the single point method.

### XBJ Enhanced the Survival Rate in MRSA-Induced Sepsis Mouse Model

**Figure 2:**
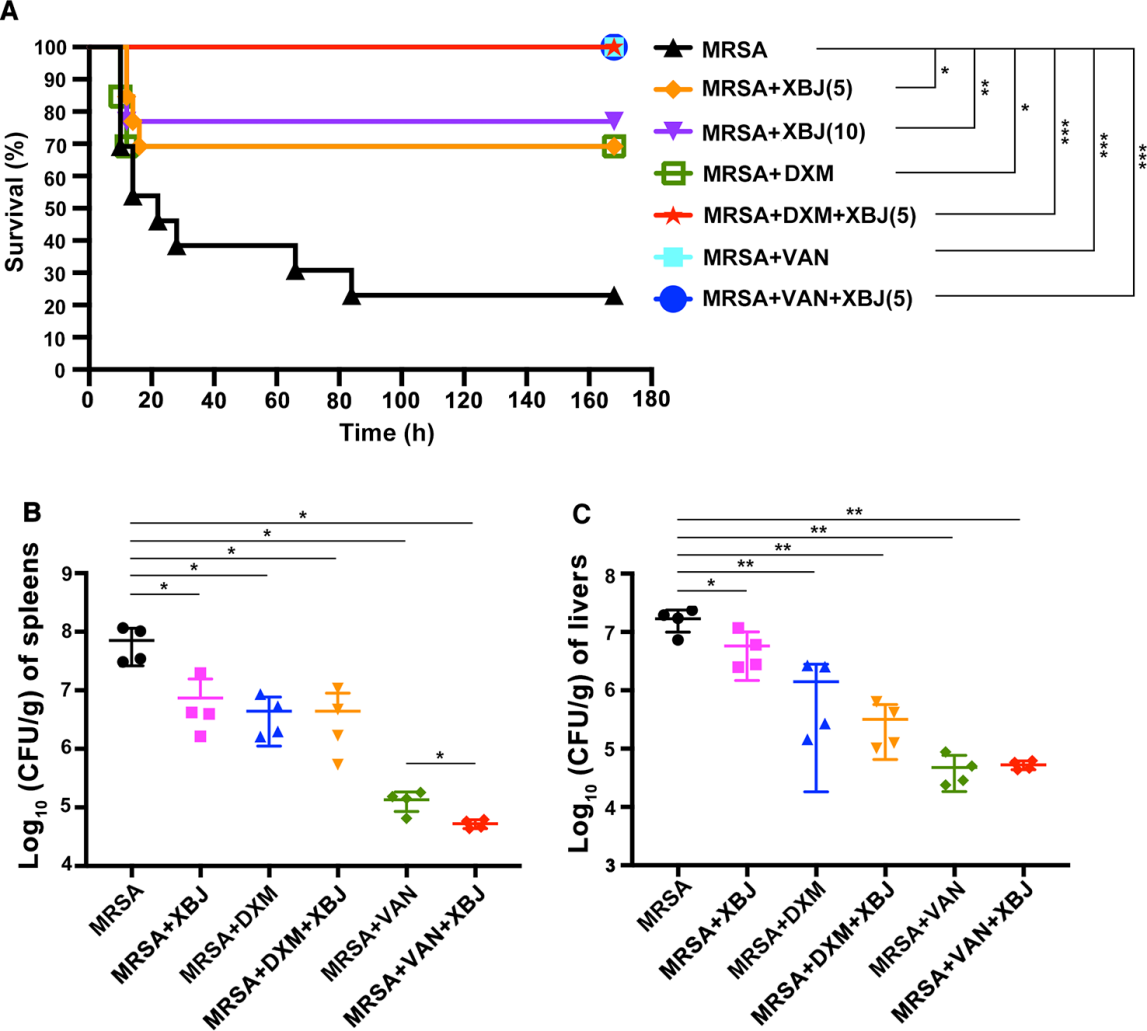
XBJ protected mice from lethal infection with MRSA and decreased the bacterial load in spleens and livers in MRSA-challenged mice. **(A)** C57BL/6J male mice were randomly divided into 7 groups (N=10–13/group). All groups of mice were intraperitoneally injected by lethal dose of MRSA (7 × 10^8^ CFU/mouse) and corresponding agents, respectively. The survival data were recorded in the following 7 days. **(B, C)** C57BL/6J female mice were randomly divided into six groups (N=4/group). All groups of mice were intraperitoneally injected with MRSA (2 × 10^8^ CFU/mouse) and XBJ (5 ml/kg), DXM (7 mg/kg), VAN (110 mg/kg), and their combination in corresponding groups. After 12 hours, mice were sacrificed and organ samples were harvested, weighed, homogenized, and coated on agar plates. After incubation at 37°C for 18 hours, CFU were calculated and the bacterial load was expressed as log_10_ (CFU/g) per organ. Data were analyzed using log-rank test **(A)** and unpaired *t*-test **(B, C)** with GraphPad Prism 8.0. *^,^**^,^***Significantly different at p < 0.05, p < 0.01, and p < 0.001, respectively.

To explore whether XBJ plays a protective role against MRSA-induced sepsis, we carried out a survival test in a mouse model of sepsis. As shown in **Figure 2A**, MRSA-induced sepsis mouse model was successfully established. The survival rate of sepsis mouse model was approximately 23%. The dosage of XBJ at 10 ml/kg was equal to the daily dose in the clinic (50 ml/person for the treatment of sepsis) (Reagan-Shaw et al., 2008). In MRSA-induced sepsis mouse model, XBJ treatment alone had protective roles at 5 and 10 ml/kg with survival rates of 69% and 77% respectively (compared with MRSA group, p < 0.05 and p < 0.01 respectively). Since there was no significant statistical difference between the survival rates of these doses of XBJ treatment, 5 ml/kg of XBJ was used in subsequent experiments. Our results showed that VAN treatment alone or combined use of XBJ and VAN could protect mice from MRSA-induced death (100% survival rate) (compared with MRSA group, p < 0.001). The survival rate of mice treated with DXM was 69% (compared with MRSA group, p < 0.05), with the same survival rate as those in XBJ (5 ml/kg) treatment group. Surprisingly, the combination of XBJ and DXM had an amazing protective effect (100% survival rate) against sepsis that was induced by a lethal dose of MRSA (compared with MRSA group, p < 0.001).

### Effects of XBJ on Decreasing the Bacterial Load in Livers and Spleens

The amount of bacteria *in vivo* is associated with the severity of sepsis. Spleens, livers, and kidneys are the most vulnerable organs during sepsis. Thus, the bacterial burdens in these organs were examined. As illustrated in **Figure 2B**, the total amount of bacteria colonies was significantly higher in spleens of sepsis model mice (7.77 ± 0.3 Log_10_ CFU/g). Compared with MRSA group, bacterial count in spleens was significantly reduced in the treatment with XBJ at dose of 5 ml/kg (6.68 ± 0.45 Log_10_ CFU/g), DXM (6.54 ± 0.34 Log_10_ CFU/g), XBJ + DXM (6.41 ± 0.56 Log_10_ CFU/g), VAN (5.1 ± 0.2 Log_10_ CFU/g), and XBJ + VAN (4.72 ± 0.07 log_10_ CFU/g). Moreover, the combined treatment group (XBJ + VAN group) demonstrated a significantly decreased bacterial load in spleens compared with VAN treatment (p < 0.05) (**Figure 2B**). Similarly, the bacterial burden in livers was significantly reduced in all treatment groups (**Figure 2C**). It was examined that XBJ did not reduce bacterial load in kidneys (data not shown). Taken together, the results illustrated that the treatment of XBJ could decrease bacterial load in spleens and livers, and the combination of XBJ with VAN provided combined effect in decreasing bacterial load in spleens.

To exclude the potential bactericidal role of XBJ on MRSA directly, the test of minimum inhibitory concentrations (MICs) was carried out *in vitro* by agar dilution method. MICs are defined as the lowest concentration of a reagent that inhibits the visible growth of a microorganism after incubation overnight (Cos et al., 2006). The results showed that all MICs data of quality control strains of bacteria to four examined antibiotics were within the standard recommended by the Clinical Laboratory and Standards Institute (CLSI) (**Supplementary Figures S1B–E** **and** **Table S2**). It indicated that the results of MICs were credible. Then, MICs of eight clinical strains of bacteria to XBJ were tested. The examined strains of bacteria were from the clinic, including *Escherichia coli*, *Pseudomonas aeruginosa*, *Klebsiella pneumoniae*, carbapenem-resistant *Klebsiella pneumoniae*, hypervirulent *Klebsiella pneumoniae*, MRSA, and two strains of *S. aureus*. As we can see from **Supplementary Figure S1G**, all colonies of bacteria grew on these agar plates containing different concentrations of XBJ. It indicated that XBJ had no antibacterial effect on these bacteria within the examined concentrations (0.1–100 μl/ml).

### The Serum Cytokine Levels Were Decreased by XBJ in MRSA-Induced Sepsis Mice

**Figure 3:**
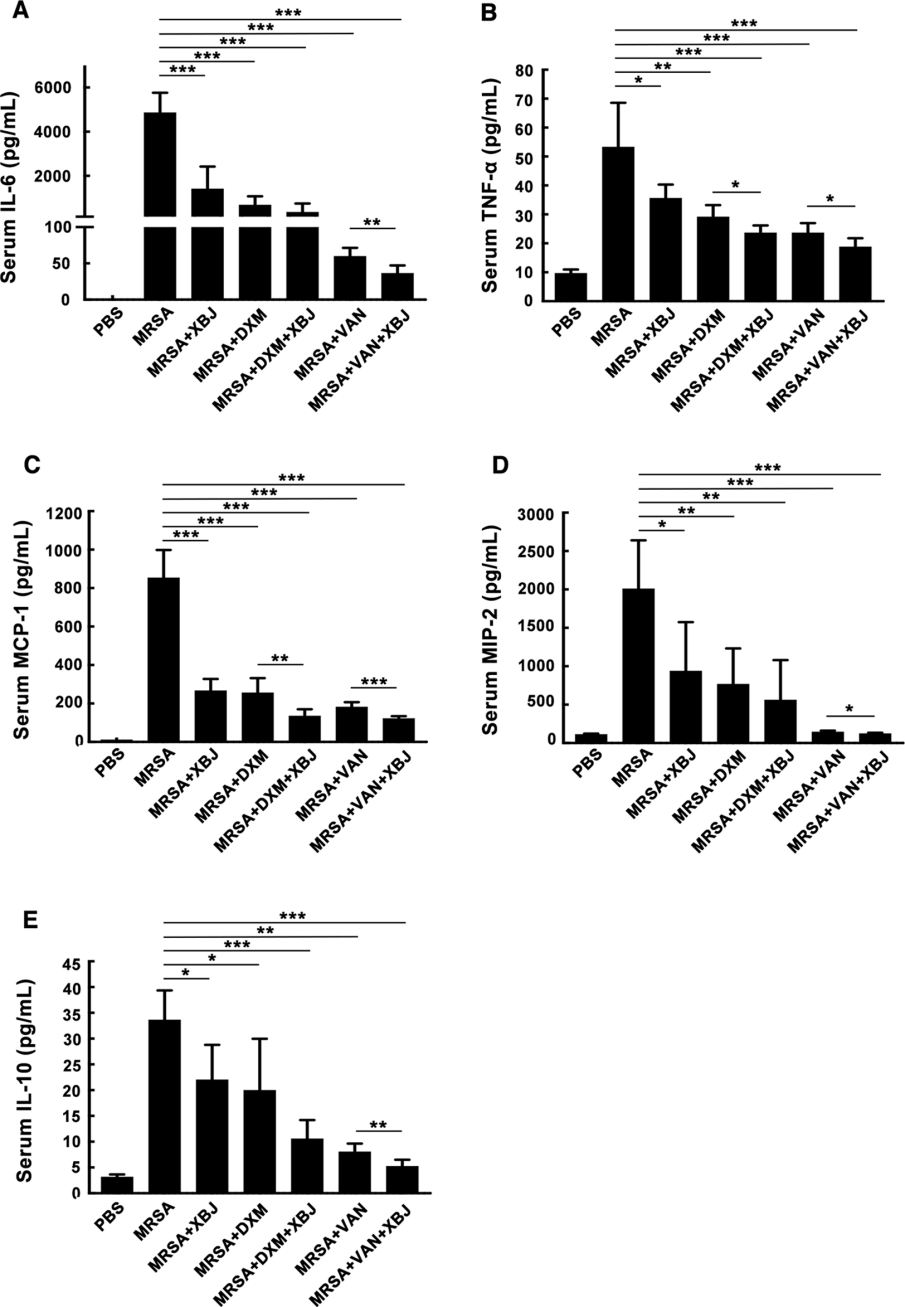
XBJ decreased the inflammatory response in MRSA-infected mice. C57BL/6J female mice were randomly divided into seven groups (N=6/group). All groups of mice were intraperitoneally injected with MRSA (4 × 10^8^ CFU/mouse) and XBJ (5 ml/kg), DXM (7 mg/kg), VAN (110 mg/kg), and their combination in corresponding groups. After 12 hours, blood samples were collected from eyeball, standing still at 4℃ for 3 hours, and then centrifuged for 25 minutes (3500 rpm, 4°C) to obtain sera. The concentrations of IL-6 **(A)**, TNF-α **(B)**, MCP-1 **(C)**, MIP-2 **(D)**) and IL-10 **(E)** were detected by ELISA. Data were shown as mean ± SD of six mice per group. *^,^**^,^***Significantly different at p < 0.05, p < 0.01, and p < 0.001, respectively.

Organ failure caused by exacerbated systemic inflammatory response is the major cause of early death in septic patients (Venet and Monneret, 2018). As demonstrated in **Figure 2**, XBJ protected septic mice from death within 20 hours after intraperitoneal injection of a lethal dose of MRSA. Therefore, we focused on XBJ-mediated attenuation of the secretion of cytokines. Among them, pro-inflammatory cytokines (IL-6, TNF-α) and chemokines (MCP-1, MIP-2) are representative mediators released in response to bacterial infection (Chousterman et al., 2017). On the other hand, IL-10 is a typical anti-inflammatory factor. In this study, the levels of IL-6, TNF-α, MCP-1, MIP-2, and IL-10 were measured in sera of mice challenged by MRSA at 12 h. As shown in **Figures 3A–E**, the levels of serum IL-6, TNF-α, MCP-1, MIP-2, and IL-10 were decreased by XBJ treatment compared to those of MRSA-challenged septic mice. It is worth noting that in the combinational treatment group (XBJ +VAN or XBJ +DXM group), the secretion of TNF-α and MCP-1 in sera was significantly inhibited compared with the mice from VAN or DXM treatment group. Moreover, the secretion of IL-6, MIP-2, and IL-10 in XBJ + VAN group was lower than those in VAN group. Taken together, the results showed that XBJ regulated the secretion of cytokines during the infection of MRSA. Furthermore, the combinational use of XBJ with VAN or DXM provided combined effects to down-regulate the inflammatory response.

### XBJ Protected Mice Against Lung, Liver, and Kidney Damage in MRSA-Challenged Mouse Model

**Figure 4:**
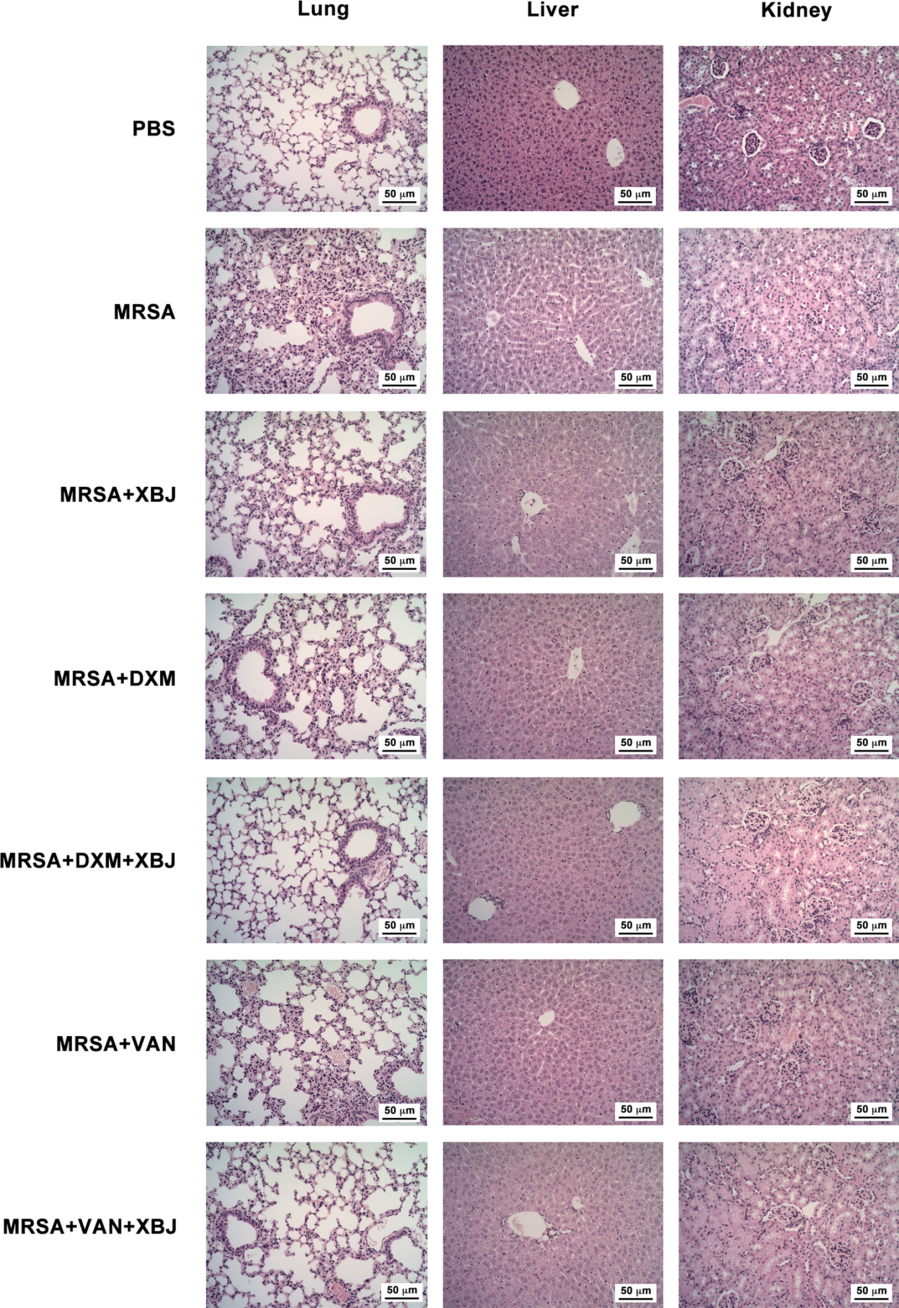
XBJ relieved organ damage caused by MRSA infection. C57BL/6J female mice were randomly divided into seven groups and intraperitoneally injected with MRSA (4 × 10^8^ CFU/mouse) and XBJ (5 ml/kg), DXM (7 mg/kg), VAN (110 mg/kg), and their combination in corresponding groups. H&E-staining (200×) of tissue sections from lungs, livers, and kidneys were carried out from the indicated groups (N = 3/group).

In sepsis, pathogenic bacteria and the drastic production of proinflammatory cytokines and chemokines contribute to the pathogenesis of multiple organ failure (acute lung, liver, and kidney injury, etc.) (Chousterman et al., 2017). To examine the protective effect of XBJ on organ damage, we conducted a histologic examination of lung, liver, and kidney. H&E-stained lungs in PBS group exhibited a normal histological structure without pathological changes (**Figure 4**). Upon MRSA infection (4 × 10^8^ CFU/mouse), lungs of the septic model mice showed obvious pathologic changes, including excessive infiltration of inflammatory cells, serous exudation in the alveolar cavity, and an increased thickness of the lung interstitium. The pathological changes observed in mice treated with XBJ, DXM, and VAN separately were less severe than in that MRSA group, showing less infiltration of inflammatory cells and clearer and thinner lung interstitium (**Figure 4**). Significantly, the combined treatment of XBJ + DXM or XBJ + VAN offered combination protective roles on lung injury in MRSA-induced sepsis mice (**Figure 4**). Similarly, the protective role of XBJ on the liver or kidney was also detected. Meanwhile, XBJ exhibited combination protection of alleviating liver or kidney damage with VAN or DXM.

### XBJ Reduced the Secretion of IL-6 and TNF-α and Exhibited Combined Effects With VAN or DXM in HK-MRSA-Stimulated Macrophages

*In vivo*, XBJ demonstrates an anti-inflammatory and protective role. Therefore, we wondered how XBJ exerted its immune regulatory effects and its target cells. It's well-known that macrophages are considered as the main contributors of inflammatory cytokines during sepsis. The *in vitro* experiments were carried out using macrophages. To exclude the possibility of inhibition on cellular viability, a cell proliferation assay was carried out in mouse macrophages cell line Raw264.7. XBJ (within 100 μl/ml) did not affect the viability of Raw264.7 within 72 h (**Supplementary Figure S2**). Thus, the highest concentration of XBJ at 100 μl/ml was considered as the safe dose for macrophages, etc.

**Figure 5:**
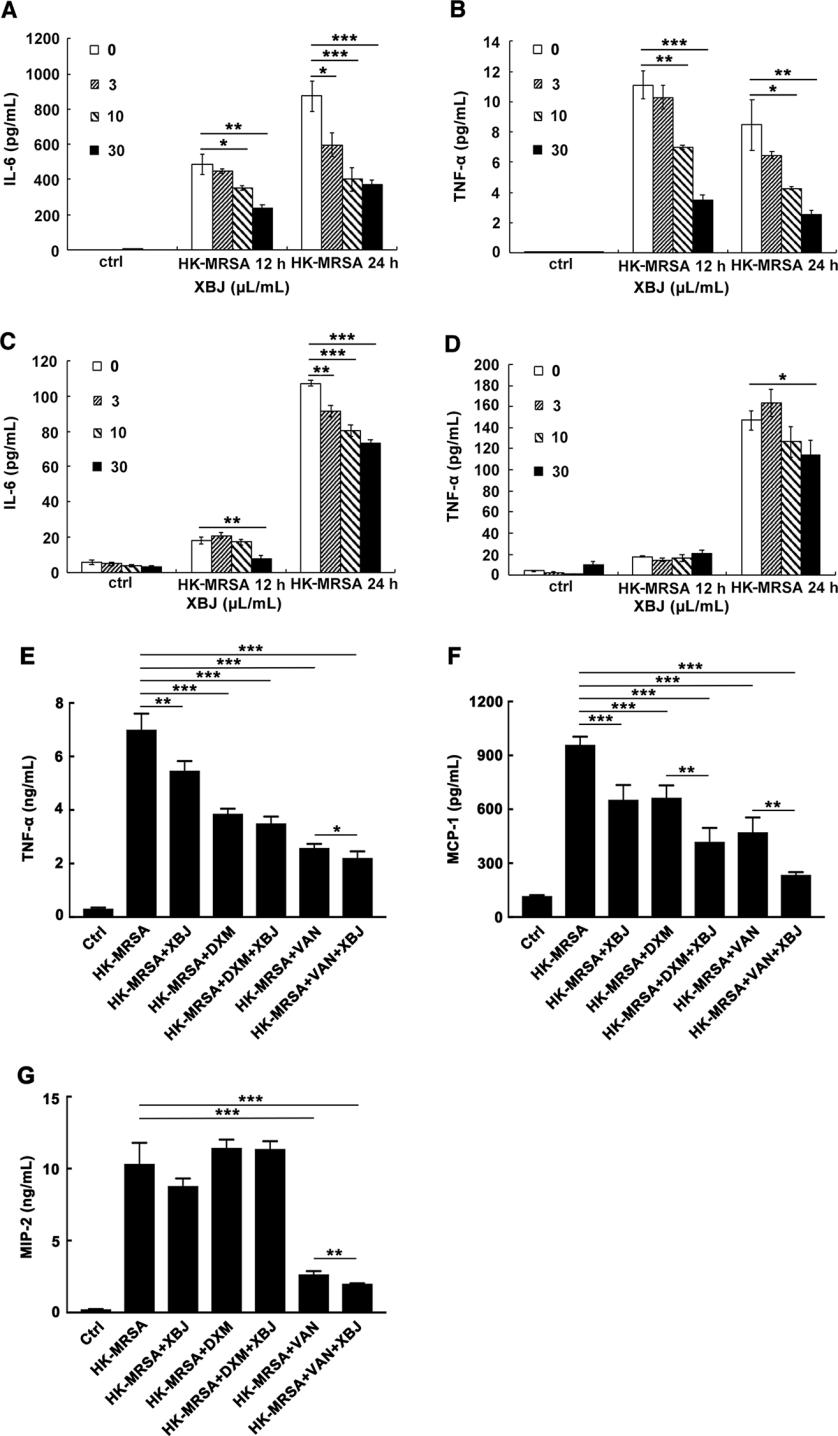
XBJ reduced the secretion of IL-6 and TNF-α and exhibited combined effects with VAN or DXM in HK-MRSA-stimulated macrophages. **(A–D)**. Mouse primary peritoneal macrophages (3.5 × 10^5^ cells/well) or iBMDMs (2 × 10^5^ cells/well) were seeded in 24-well plates and incubated overnight. Cells were stimulated by HK-MRSA (MOI=10) and treated with different concentrations of XBJ for 12 or 24 hours. The secretion of cytokines in the cell culture supernatants were determined by ELISA. The expression of IL-6 and TNF-α were detected in primary peritoneal macrophages **(A**, **B)** or in iBMDMs **(C**, **D)**. Data were shown as mean ± SD (n=3). **(E–G)** XBJ+DXM or XBJ+VAN decreased the production of HK-MRSA-induced inflammatory mediators in Raw264.7. Raw264.7 (2 × 10^5^ cells/well) were seeded in 24-well plates and incubated overnight. Cells were stimulated by HK-MRSA (MOI=10) and treated with XBJ (10 µl/ml), DXM (7 µM), VAN (15 µM), and their combination for 24 hours. The secretion of TNF-α **(E)**, MCP-1 **(F)**, and MIP-2 **(G)** in the supernatants were determined by ELISA. Data were shown as mean ± SD (n=4). *^,^**^,^***Significantly different at p < 0.05, p < 0.01, and p < 0.001, respectively.

MRSA was heat-killed/inactivated to maintain the structure of bacteria and the stimulatory bioactivity of PAMPs without viability which was verified by agar plate culture. Macrophages were then stimulated by HK-MRSA to elicit an inflammatory response, and the regulatory roles of XBJ on the secretion of inflammatory cytokines were examined. Data showed that XBJ decreased the production of pro-inflammatory cytokines IL-6 and TNF-α in a dose-dependent manner in mouse primary macrophages (**Figures 5A, B**). XBJ also inhibited the secretion of IL-6 and TNF-α after HK-MRSA stimulation in iBMDMs (**Figures 5C, D**). In conclusion, XBJ down-regulates HK-MRSA-induced inflammatory response.

The combined anti-inflammatory effects of XBJ+DMX or XBJ+VAN were also illustrated in HK-MRSA-stimulated Raw264.7. The dosages of DXM and VAN were calculated according to clinical dose and our experiments *in vivo*. As shown in **Figure 5**, the levels of TNF-α, MCP-1, and MIP-2 were significantly decreased by XBJ+VAN treatment compared with VAN treatment alone. The level of MCP-1 in XBJ+DMX group was lower than that in DXM group. Unexpectedly, the concentration of IL-6 was too low to be detected in each group. Taken together, the results showed that the combinational use of XBJ with VAN or DXM provided combined anti-inflammatory function, in correspondence with the combinational role observed *in vivo* (**Figure 3**).

### XBJ Suppressed the Production of Inflammatory Mediators Evoked by PAMPs Mimicking *S. aureus* Infection in Raw264.7 and Primary Macrophages

**Figure 6:**
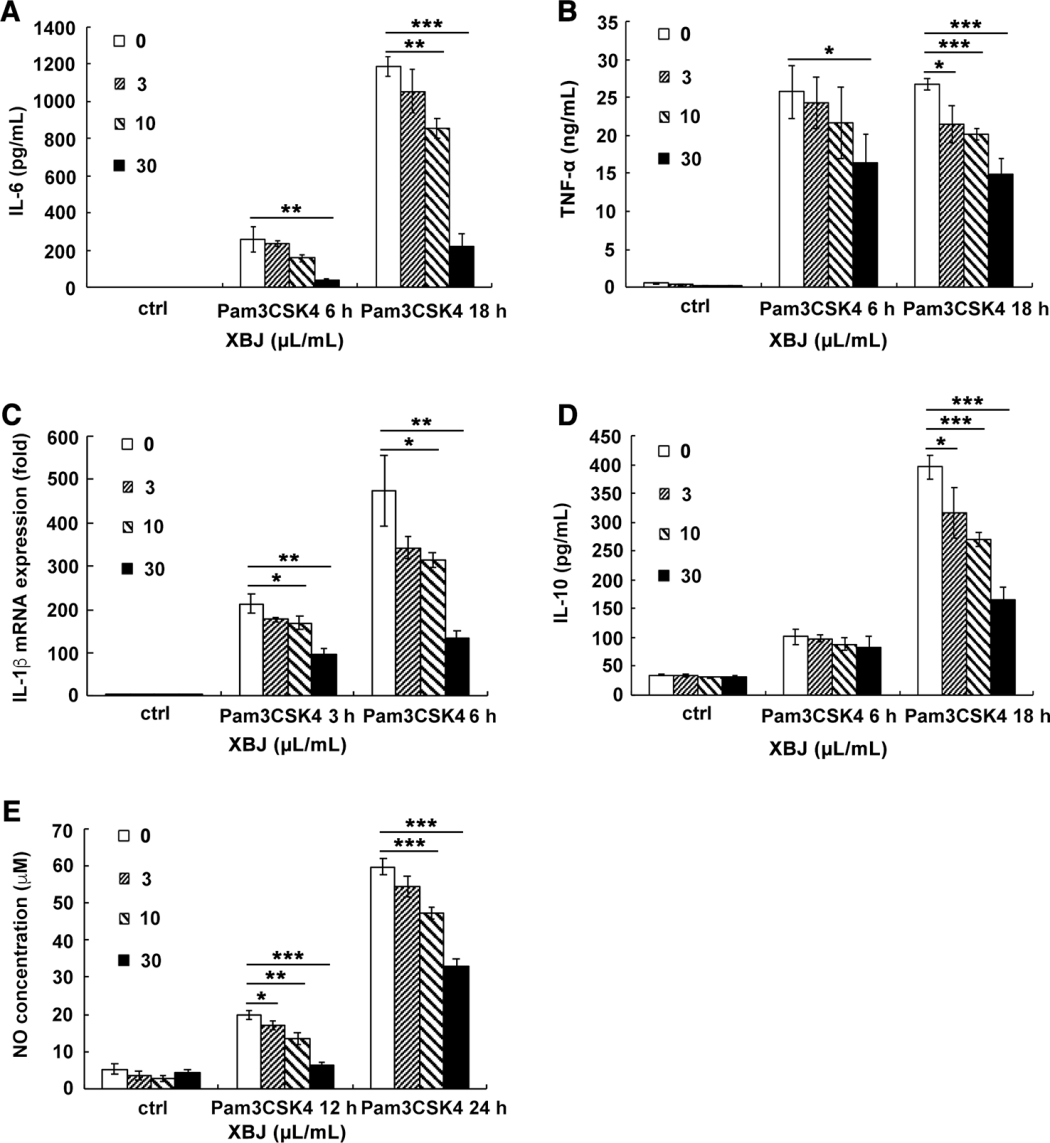
XBJ inhibited the production of Pam3CSK4-induced inflammatory mediators in Raw264.7. Raw264.7 cells were seeded (2 × 10^5^ cells/well) in 24-well plates overnight and stimulated by Pam3CSK4 (100 ng/ml) with different concentrations of XBJ for 6 or 18 hours. Cell culture supernatants were collected and the concentrations of IL-6 **(A)**, TNF-α **(B)**, and IL-10 **(D)** were measured by ELISA. IL-1β **(C)** mRNA expression was examined by qRT-PCR. NO concentrations **(E)** was measured by Griess assay at 12 or 24 hours after stimulation. Data were shown as mean ± SD (n=3). *^,^**^,^***Significantly different at p < 0.05, p < 0.01, and p < 0.001, respectively.

**Figure 7:**
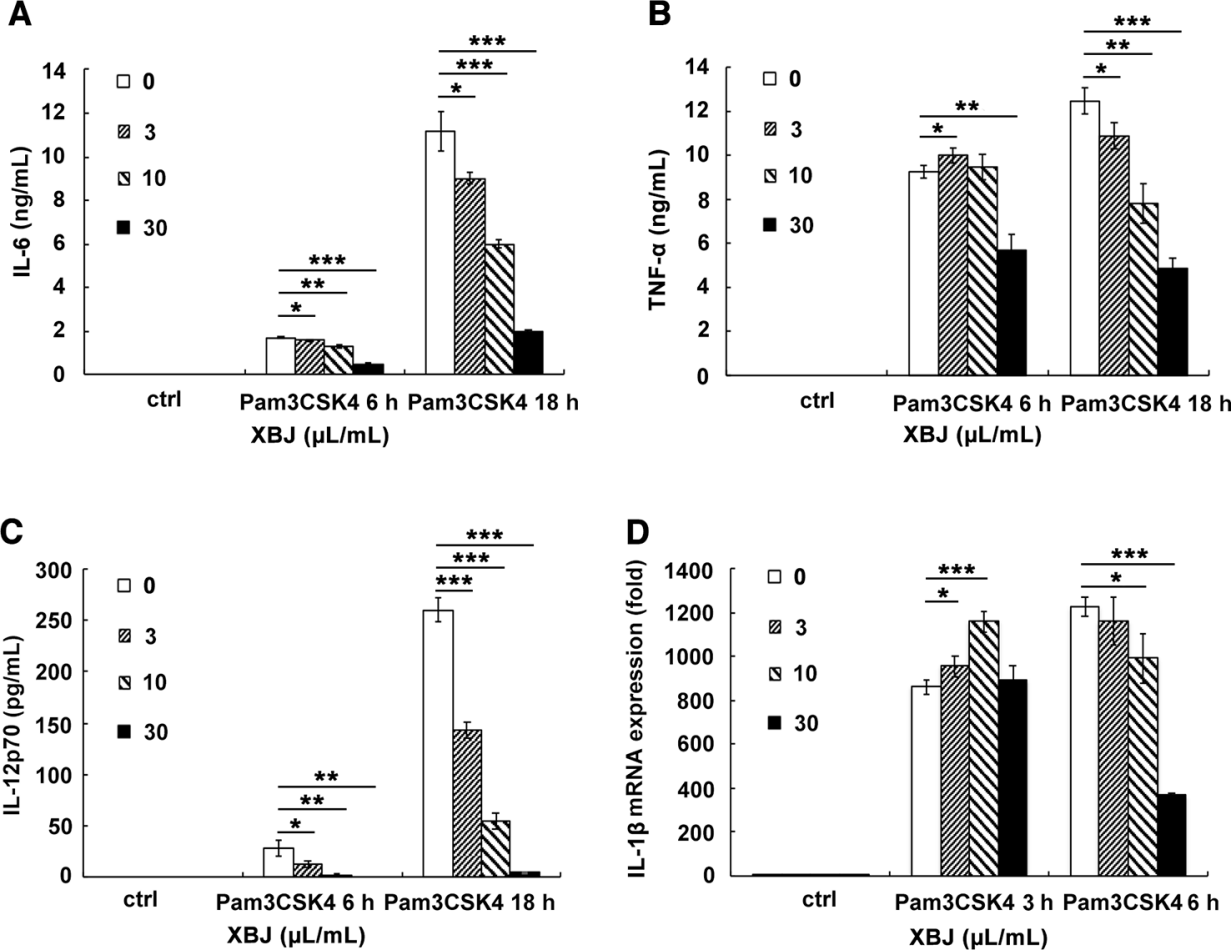
XBJ decreased the production of Pam3CSK4-induced IL-6, TNF-α, IL-1β, and IL-12 in mouse primary macrophages. Mouse primary peritoneal macrophages were seeded at 3.5 × 10^5^ cells/well in 24-well plates and cultured overnight. Cell were treated with a range of concentration of XBJ with or without Pam3CSK4 (100 ng/ml) for 3, 6, or 18 hours. The concentrations of IL-6 **(A)**, TNF-α **(B)**, and IL-12p70 **(C)** in the cell culture supernatant were detected by ELISA. And the expression of IL-1β **(D)** mRNA was examined by qRT-PCR. Data were shown as mean ± SD (n=3). *^,^**^,^***Significantly different at p < 0.05, p < 0.01, and p < 0.001, respectively.

PAMPs are highly conserved structures of pathogens and identified by PRRs on immune cells, which leads to an innate immune response. In the case of *S. aureus* infection, PGN and lipoprotein, the main components of the bacterial cell wall, stimulates TLR2 as PAMPs. Pam3CSK4 is a synthetic tripalmitoylated lipopeptide that mimics the acylated amino terminus of bacterial lipoproteins. Thus, it is usually used as a mimicking stimulator of bacterial infection *in vitro*. The stimulatory cell models were set up with the stimulation of Pam3CSK4 or PGN to explore the anti-inflammatory role of XBJ. Our results demonstrated that XBJ decreased mRNA expression of IL-6, TNF-α, MCP-1, and MIP-2 in a dose-dependent manner in Pam3CSK4-stimulated Raw264.7 (**Supplementary Figure S3**). Meanwhile, high doses of XBJ were needed to inhibit the expression of these inflammatory cytokines in PGN-stimulated cell model (**Supplementary Figure S4**). So, Pam3CSK4 was chosen as a stimulator in the following *in vitro* experiments. IL-6, TNF-α, IL-1β, and IL-12 are hallmark inflammatory mediators of sepsis that constitute the cytokine storm. As shown in **Figure 6**, XBJ decreased the production of IL-6, TNF-α, IL-1β, NO, and even anti-inflammatory IL-10 in Raw264.7. A similar anti-inflammatory role of XBJ was observed in mouse primary peritoneal macrophages (**Figure 7**). Taken together, our results have demonstrated that XBJ down-regulates Pam3CSK4-induced inflammatory response by decreasing the production of pro-inflammatory cytokines and IL-10.

### XBJ Inhibited Pam3CSK4-Induced Inflammatory Response *Via* NF-κB and MAPK Pathways in Mouse Primary Macrophages

**Figure 8:**
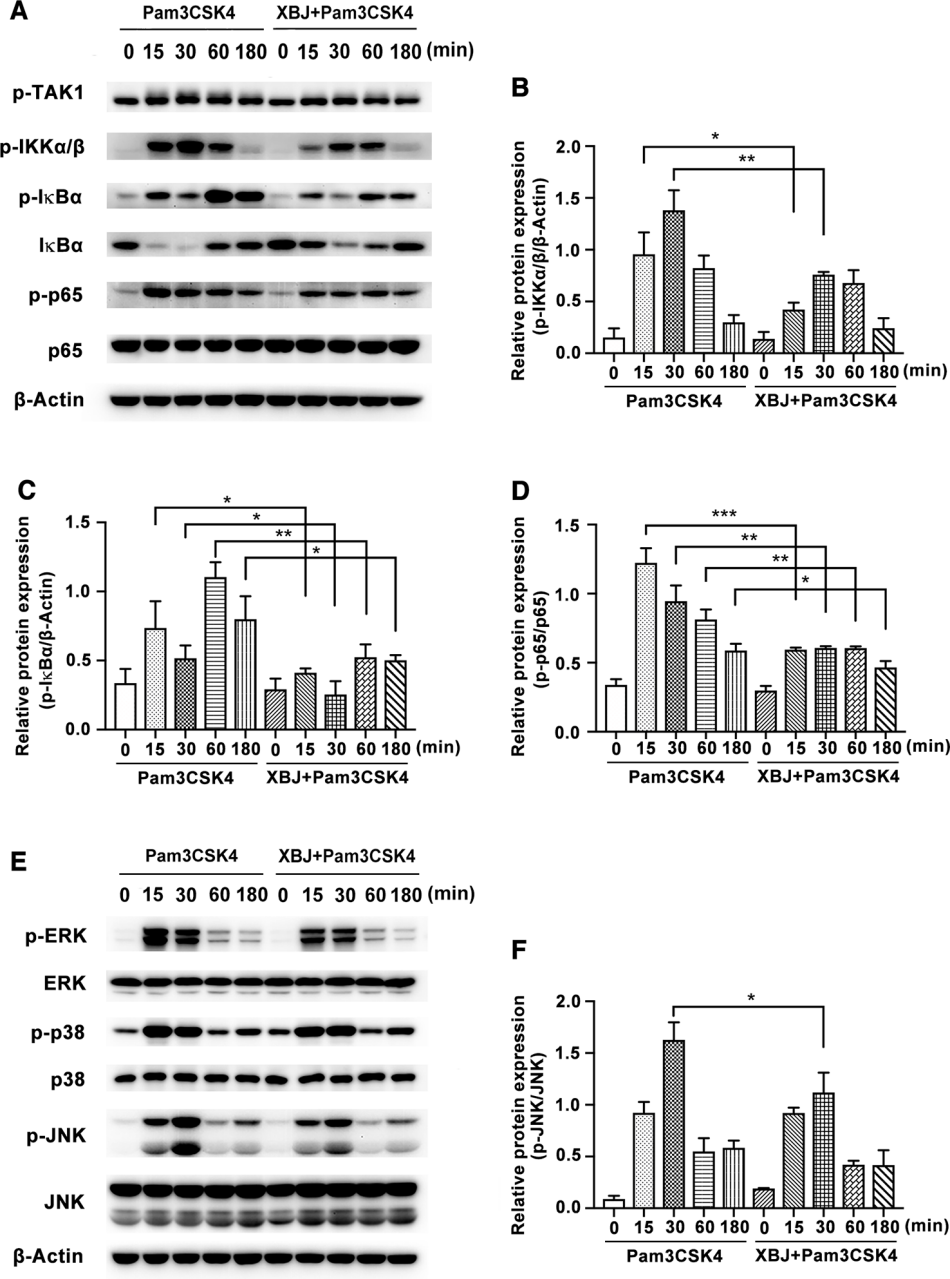
XBJ inhibited Pam3CSK4-induced activation of NF-κB and MAPK pathways. Mouse primary peritoneal macrophages (1× 10^6^ cells/well) were plated overnight, and then stimulated with Pam3CSK4 (100 ng/ml) in the presence or absence of XBJ (10 μl/ml) for different time periods. **(A, E)** The phosphorylation of signal proteins of NF-κB and MAPK pathway were examined by Western blot with the reference of the corresponding total proteins. The bar graphs showed the statistical results for the relative quantitative expression of p-IKKα/β **(B)**, p-IκBα **(C)**, p-p65 **(D)** and p-JNK **(F)** expression. Data were shown with the means ± SD of at least three independent experiments. *^,^**^,^***Significantly different at p < 0.05, p < 0.01, and p < 0.001, respectively.

Next, the molecular mechanisms of the anti-inflammatory effects of XBJ were further explored. After ligation of Pam3CSK4, myeloid differentiation factor 88 (MyD88) is recruited to TLR2, and a complex of IL-1R-associated kinases (IRAKs) and TNFR-associated factor 6 (TRAF6) is subsequently formed. After that, ubiquitinated TRAF6 activates a complex of TGF-β-activated kinase 1 (TAK1) and TAK1-binding protein, resulting in the phosphorylation of NF-κB essential modulator and the activation of IκB kinase (IKK) complex composed of IKKα and IKKβ. Then, phosphorylated IκB is degraded, and released NF-κB translocates to the nucleus where it drives the expression of various proinflammatory cytokines, etc. (Takeuchi and Akira, 2010). Simultaneously, phosphorylated TAK1 activates MAPK cascades, leading to the activation of extracellular signal regulated kinase (ERK), c-Jun N-terminal kinase (JNK), and p38 MAPK pathways, which are also critical for the induction of cytokine genes. As demonstrated in **Figures 8A–D**, Pam3CSK4 triggered obvious activation of NF-κB pathway, while XBJ decreased the phosphorylation of IKKα/β, IκBα, and p65 NF-κB. Treatment with Pam3CSK4 provoked a rapid increase in the phosphorylation of ERK, JNK, and p38 in mouse primary macrophages, while the phosphorylation of JNK was down-regulated by XBJ (**Figures 8E, F**). Taken together, XBJ influences the activation of NF-κB and MAPK pathways in Pam3CSK4-stimulated macrophages.

### XBJ Inhibits PI3K/Akt Phosphorylation in Pam3CSK4-Induced Mouse Primary Macrophages

**Figure 9:**
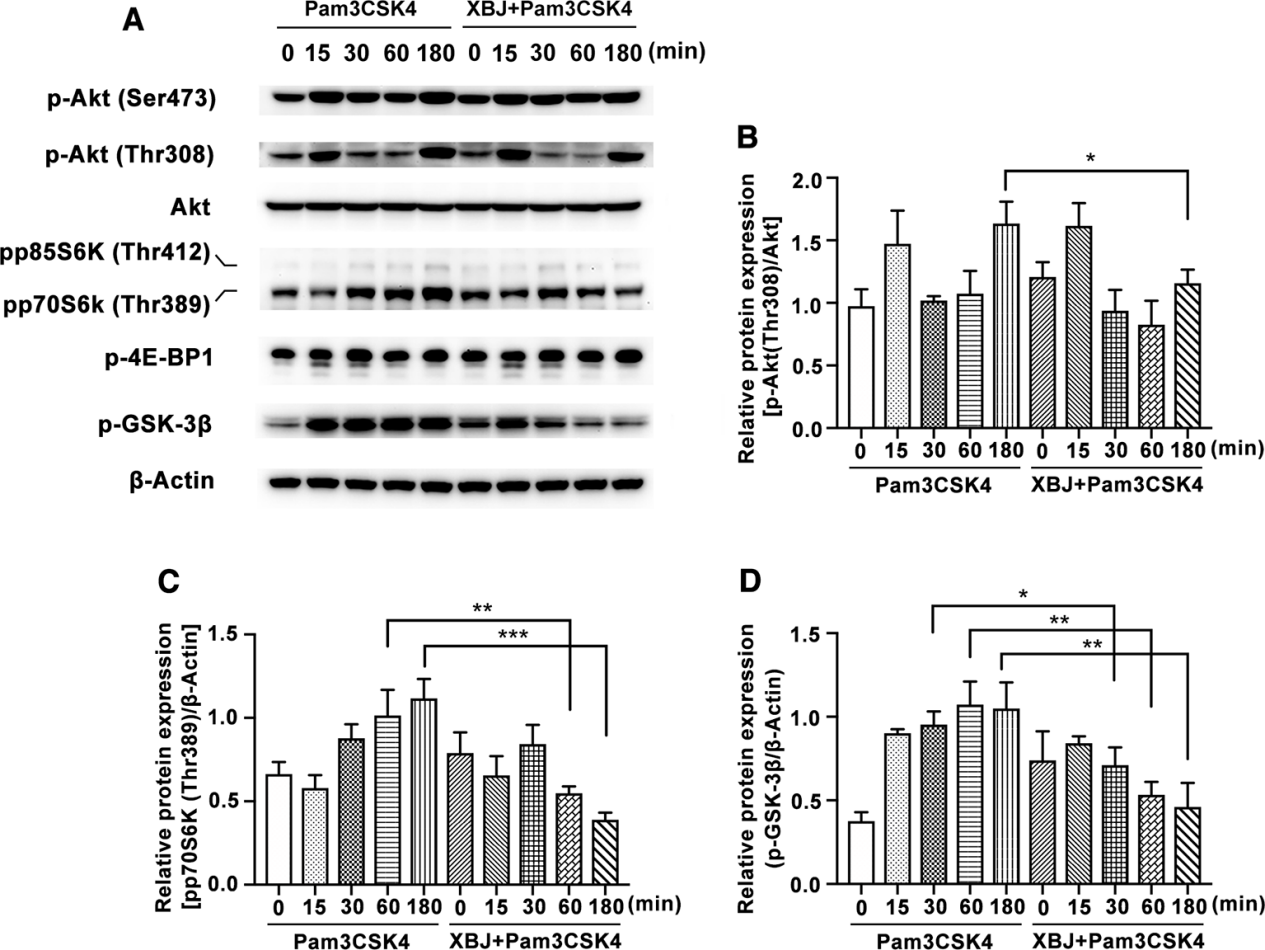
XBJ inhibited Pam3CSK4-induced activation of PI3K/Akt pathway. Mouse primary peritoneal macrophages (1× 10^6^ cells/well) were plated overnight, and then stimulated with Pam3CSK4 (100 ng/ml) in the presence or absence of XBJ (10 μl/ml) for different time periods. **(A)** The phosphorylation of signal proteins of PI3K/Akt pathway were examined by Western blot with the reference of total Akt and β-Actin. The bar graphs showed the statistical results for the relative quantitative expression of p-Akt (Thr308) **(B)**, pp70S6K (Thr389) **(C)**, and p-GSK-3β (Ser9) **(D)**. Data were shown with the means ± SD of at least three independent experiments. *^,^**^,^***Significantly different at p < 0.05, p < 0.01, and p < 0.001, respectively.

The PI3K/Akt/mammalian target of rapamycin (PI3K/Akt/mTOR) signaling pathway has emerged as a critical pathway of TLRs in coordination with the inflammatory response (Li et al., 2018). PI3K pathway is widely expressed and participates in various biological functions. Recognized as one of the key downstream proteins of PI3K, Akt is a well-known evolutionarily highly conserved serine/threonine protein kinase. The sites of Ser473 and Thr308 are typical phosphorylated sites of Akt. Following the stimulation of Pam3CSK4, Akt is phosphorylated by PI3K activation and promotes the phosphorylation of mTOR. This enhances the initiation of gene translation partially *via* phosphorylation of eukaryotic initiation factor 4E-binding protein 1 (4E-BP1) and proteins of p70S6 kinase (p70S6K). As **Figures 9A–C** shown, XBJ inhibits Pam3CSK4-triggered phosphorylation of Akt (Thr308) and p70S6K (Thr389).

Glycogen synthase kinase (GSK)-3 is a critical downstream element of PI3K/Akt cell survival pathway which activity can be inhibited by Akt-mediated phosphorylation of Ser21 on GSK-3α and Ser9 on GSK-3β. PI3K/Akt/GSK-3β signaling pathway contributes to the production of IL-10 after TLR2 ligation (Saraiva and O'Garra, 2010). Increasing evidence showed that GSK-3β inactivation has a potential therapeutic role in the control of infective diseases (Wang et al., 2014). XBJ decreases the phosphorylation of GSK-3β in mouse primary macrophages (**Figures 9A, D**), which may account for the down-regulated IL-10 expression by XBJ treatment.

### XBJ Has Better Anti-Inflammatory Effect Than Its Main Components (HSYA and PAE) or the Combination of Main Components (HSYA+PAE) in Pam3CSK4-Stimulated Mouse Primary Macrophages

**Figure 10:**
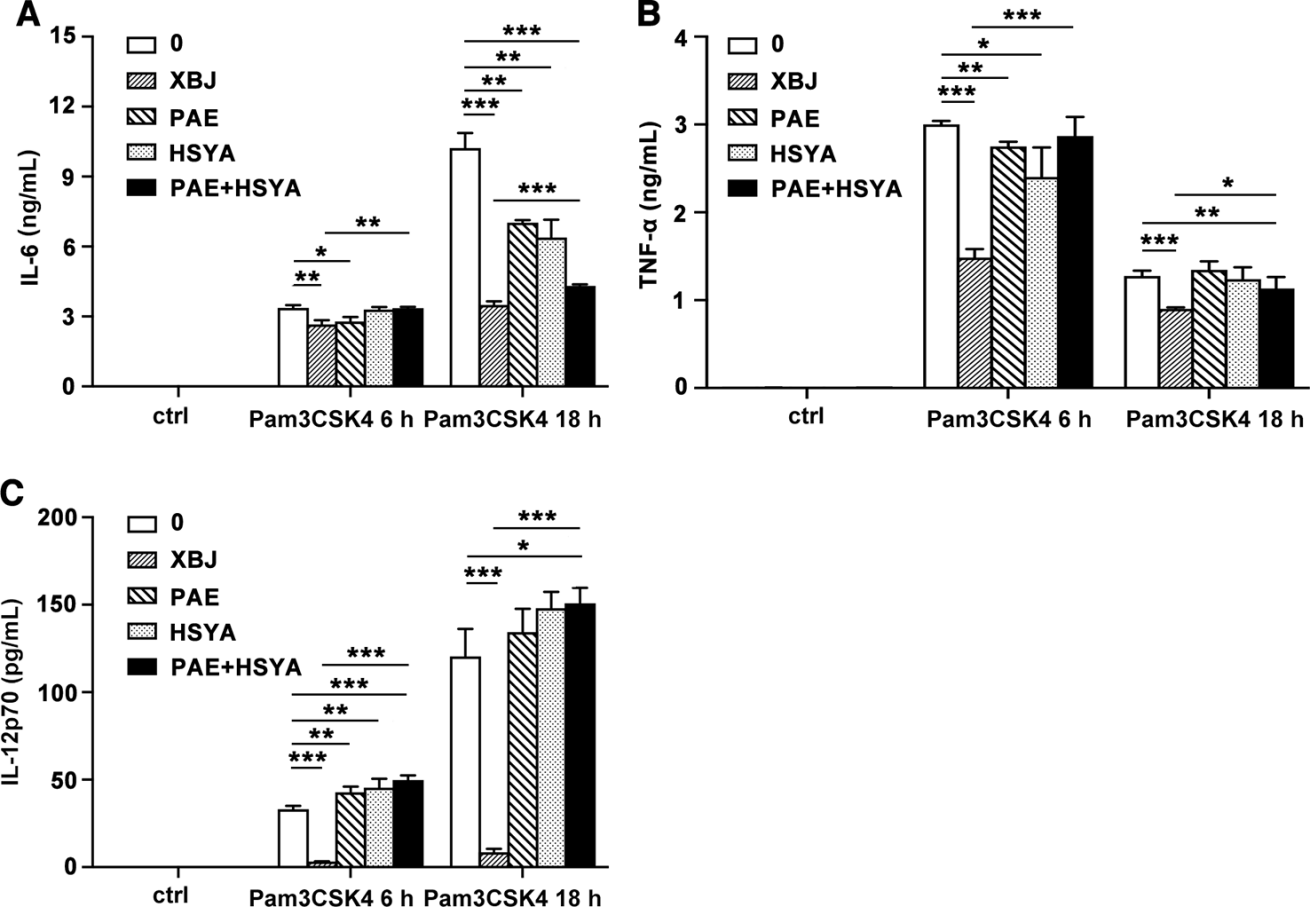
Comparison of anti-inflammatory effects of XBJ and its main components. Mouse primary peritoneal macrophages were seeded at 3.5 × 10^5^ cells/well in 24-well plates and cultured overnight. Cell were treated with XBJ (10 µl/ml), PAE (26.7 µg/ml), and HSYA (13.7 µg/ml) with or without Pam3CSK4 (100 ng/ml) for 3, 6, or 18 hours. The concentrations of IL-6 **(A)**, TNF-α **(B)**, and IL-12p70 **(C)** in the cell culture supernatant were detected by ELISA. Data were shown as mean ± SD (n=3). *^,^**^,^***Significantly different at p < 0.05, p < 0.01, and p < 0.001, respectively.

An important characteristic of TCM is to mix many bioactive active ingredients to play a multi-target regulatory or synergistic role. Among the diversified components of XBJ, the two most abundant components are hydroxysafflor yellow A (HSYA) and paeoniflorin (PAE) according to UPLC-MS/MS analysis. It was reported that HSYA effectively ameliorated sepsis-induced apoptosis of CD4^+^ T lymphocytes by its anti-inflammatory and anti-apoptotic effects. (Wang et al., 2017). On the other hand, PAE reduced macrophage activation by inhibiting TLR2/4 signaling expression in type 2 diabetic nephropathy (Zhang et al., 2017). The examined dosage of HSYA and PAE at 13.7 and 26.7 µg/ml respectively were equivalent to their concentrations in XBJ (10 µl/ml). HSYA or PAE treatment alone or their combination decreased the production of IL-6 at 18 h. However, XBJ reduced the expression of IL-6 and TNF-α more significantly than HSYA or PAE treatment alone or their combination treatment (**Figures 10A, B**). Interestingly, XBJ inhibits IL-12p70 robustly while HSYA and PAE showed no effect (**Figure 10C**).

## Discussion

Though the survival rate of septic patients is improved by life-supporting systems in intensive care units, sepsis remains a significant threat to public health. Early identification and elimination of the source of infection using antibiotics or anti-microbial peptides are the first choices to control the deterioration of sepsis. VAN was recommended as a first-line agent for MRSA infection, but there are many adverse effects following treatment with VAN, such as nephrotoxicity, hypotension, phlebitis, hypersensitive reactions, and red man syndrome, etc. Nephrotoxicity is the most serious adverse effect, with an incidence of 9.8–23% (Jeffres, 2017). Besides, increasing evidence from studies of sepsis suggests that the antibiotic-mediated release of biologically active PAMPs derived from G^+^ bacteria, Gram-negative bacteria, or fungal organisms is associated with rapid clinical deterioration (Lepper et al., 2002). Worse still, the extensive emergence of multidrug-resistant bacteria is casting a darkening shadow to anti-infective treatment. Thus, novel therapeutic strategies are urgently needed.

One of the complementary strategies is host-directed therapy (HDT), which regulates the aspect of host-pathogen interaction during infection and bypasses the problem of antibiotic resistance (Kaufmann et al., 2018). The innate immunity of the host, which is responsible for the immediate response to invading pathogens, plays a crucial role in the initiation of the pathophysiology of sepsis. The data from clinical studies of sepsis showed that the plasma concentrations of IL-1β, IL-6, IL-8, MCP-1, IL-10, and IL-4 were significantly higher in non-survivors when compared with survivors (Bozza et al., 2007; Surbatovic et al., 2015). The researchers also observed that the levels of IL-1β, IL-6, IL-10, MCP-1, and TNF-α were significantly increased in septic shock as compared with severe sepsis. It indicated that not only the high levels of pro-inflammatory cytokines but also the high levels of anti-inflammatory cytokines are related to high mortality during sepsis. Currently, anti-inflammatory treatment such as IL-1 receptor blockade, anti-IL-6, and anti-TNF are considered to be promising approaches in the early stage after the onset of sepsis, but it is too costly (Tanaka et al., 2016; Venet and Monneret, 2018). Moreover, blockade of one important cytokine is not sufficient for the treatment of a syndrome like sepsis.

Xuebijing injection (XBJ) has been used for the treatment of sepsis in China for 15 years. In our study, XBJ reduced the secretion of IL-6 and TNF-α in response to HK-MRSA in macrophages. XBJ also suppressed the production of pro-inflammatory cytokines, chemokines, and even anti-inflammatory cytokines evoked by PAMPs mimicking *S. aureus,* including PGN (the main cell wall composition of G^+^ bacteria, recognized by TLR2/6) and Pam3CSK4 (a triacyl lipopeptide, recognized by TLR2/1). Since XBJ has the downregulatory role of the inflammatory response caused by TLR2 activation, we speculate that it can also down-regulate the inflammatory response caused by pathogens containing TLR2 agonists. In MRSA-induced sepsis mouse model, XBJ decreased the secretion of IL-6, TNF-α, MCP-1, MIP-2, and IL-10 in sera and alleviated lung, liver, and kidney damage. XBJ also enhanced the survival rate in MRSA-induced sepsis mouse model. Collectively, our data indicated that XBJ has a significant anti-inflammatory activity to control the overwhelming production of multiple inflammatory mediators *in vitro* and *in vivo*. It's a more efficient way of down-regulating all kinds of cytokines compared with using individual neutralizing antibodies during bacterial infection.

What's more, the combination of XBJ with VAN or DXM exhibited combined effects in protecting mice from MRSA infection. In MRSA-infected mice, combinational use of XBJ with VAN significantly inhibited the secretion of IL-6, TNF-α, MCP-1, MIP-2, and IL-10 in sera, decreased bacterial load in spleens, and alleviated lung, liver, and kidney damage compared with VAN treatment alone. Similarly, the survival rate of the combination group of XBJ and DXM reached 100% in MRSA-infected sepsis mice. *In vitro*, XBJ+DMX or XBJ+VAN also had the combined anti-inflammatory effects in HK-MRSA-stimulated Raw264.7. Thus, XBJ can serve as adjuvant therapy in dealing with MRSA-induced sepsis.

Our results demonstrated that treatment with XBJ significantly decreased the live bacterial burdens in spleens and livers from septic mice, but XBJ had no antibacterial effect on eight clinical bacterial strains within the examined concentrations of 0.1–100 μl/ml. It indicated that the decreased bacterial count in organs by XBJ treatment did not depend on its bioactivity as an antibiotic. It suggested that XBJ might regulate host immune response to eliminate bacteria, e.g. MRSA, by promoting the phagocytosis of immune cells (macrophages, neutrophils, and dendritic cells, etc.).

Taken together, XBJ has the functions of regulating immune response, including decreasing inflammatory mediators and bacterial load. XBJ plays a protective role in infection caused by bacteria, especially antibiotic-resistant bacteria such as MRSA. It is not only the wisdom of TCM to regulate invariable innate immunity to fight against infection caused by various and frequently mutating pathogens, but also a new therapeutic strategy to deal with the problem of antimicrobial resistance.

The production of inflammatory mediators is regulated by multiple signaling pathways. Our results showed that XBJ inhibited the activation of NF-κB, MAPK, and PI3K/Akt pathways in Pam3CSK4-induced mouse primary macrophages. Previous studies have demonstrated that HSYA and PAE could alleviate the phosphorylation of NF-κB (IκBα and p65) (Li et al., 2017; Hu et al., 2018). In our study, XBJ also inhibited the phosphorylation of IKKα/β and its downstream molecules, but it did not affect the phosphorylation of TAK1. Besides, it was reported that PAE suppressed Concanavalin A-mediated phosphorylation of Akt (Ser473) in human hepatic sinusoidal endothelial cells (Gong W. G. et al., 2015). We've demonstrated that XBJ down-regulated the phosphorylation of Akt (Thr308), p70S6K (Thr389), and GSK-3β in PI3K/Akt signaling and JNK MAPK. In short, XBJ plays an anti-inflammatory role *via* NF-κB, MAPK, and PI3K/Akt pathways. Whether XBJ promotes phagocytosis needs to be determined. Our results also indicated that there are other anti-inflammatory substances besides HSYA and PAE in XBJ, so XBJ might play a better anti-inflammatory and protective role in the treatment of sepsis in the clinic than HSYA and PAE. In brief, the effects of TCM complex are not through the accumulation of functions of individual compounds and TCM has the advantage to play the role of immunoregulation.

In summary, XBJ downregulated the secretion of inflammatory cytokines, decreased bacterial load, and alleviated organ damage in MRSA-challenged sepsis mice. The combination of XBJ with VAN or DXM had combined protective effects in this model. Besides, this study also provided valuable insights into the down-regulatory actions on the activation of signaling pathways initiated by bacterial mimic Pam3CSK4. The present work dissected the protective role of XBJ in severe MRSA infection, which broadens the therapeutic role of XBJ in drug-resistant bacterial infection.

## Data Availability Statement

All datasets generated for this study are included in the article/**Supplementary Material**.

## Ethics Statement

All the experimental protocols were carried out in accordance with the National Institute of Health Guide for the Care and Use of Laboratory Animals and approved by Shanghai Public Health Clinical Center Laboratory Animal Welfare and Ethics Committee with the number of 2019-A006-01.

## Author Contributions

TL and YQ are joint first authors of the study. YZ and HA are joint senior authors of the study. YZ and HA designed the experiments. TL, YQ, ZM, PZ, TS, XJ, LP, FQ, and GY performed the study. TL, YZ, and HA analyzed the data. TL, YQ, YZ, and HA wrote the manuscript. All authors read and approved the manuscript.

## Funding

This study was supported by National Natural Science Foundation of China (81471537) and the Program for Professor of Special Appointment (Eastern Scholar) at Shanghai Institutions of Higher Education, 13th Five-Year National Science and Technology Major Project for Infectious of China (2017ZX10305501-002) and Interdisciplinary Project of “Clinical Immunology of Traditional Chinese Medicine” in Shanghai (30304113598).

## Conflict of Interest

The authors declare that the research was conducted in the absence of any commercial or financial relationships that could be construed as a potential conflict of interest.
