# Supplementary material for: Xuebijing Injection Alleviates Pam3CSK4-Induced Inflammatory Response and Protects Mice From Sepsis Caused by Methicillin-Resistant Staphylococcus aureus
Source: Front Pharmacol. 2020 Feb 21;11:104. doi: 10.3389/fphar.2020.00104 (PMC7047170; doi:10.3389/fphar.2020.00104)
Supplement: Supplementary file 1 [file DataSheet_1.pdf]

SUPPLEMENTARY MATERIAL

Xuebijing Injection Alleviates Pam3CSK4-induced Inflammatory Response and Protects Mice from Sepsis Caused by Methicillin-resistant *Staphylococcus aureus*

Tiantian Li[1, †], Yiming Qian[2, †], Zhulei Miao[1], Peiyong Zheng[3], Ting Shi[1], Xinru Jiang[1], Lingyun Pan[4], Fenghua Qian[2], Guizhen Yang[1], Huazhang An[5,*], Yuejuan Zheng[1,*]

[1] Department of Immunology and Microbiology, School of Basic Medical Sciences, Shanghai University of Traditional Chinese Medicine, Shanghai 201203, China

[2] Department of Emergency, Yueyang Hospital of Integrated Traditional Chinese and Western Medicine, affiliated to Shanghai University of Traditional Chinese Medicine, Shanghai 200437, China

[3] Institute of Digestive Diseases, Longhua Hospital affiliated to Shanghai University of Traditional Chinese Medicine, Shanghai 200032, China

[4] Experiment Center for Science and Technology, Shanghai University of Traditional Chinese Medicine, Shanghai 201203, China

[5] Clinical Cancer Institute, Center of Translational Medicine, Second Military Medical University, Shanghai 200433, China

***Correspondence:**

Yuejuan Zheng

13641776412@163.com or zhengyj@shutum.edu.com

Huazhang An

anhz@immunol.org

[†] These authors have contributed equally to this work.

Materials and Methods

Reagents

Mueller-Hinton agar was purchased from Thermo Fisher Scientific (Waltham, MA, USA). Levofloxacin, meropenem, vancomycin and oxacillin were purchased from National Institutes for Food and Drug Control (Beijing, China).

Minimum Inhibitory Concentrations Testing

Two-fold serial dilution method was adopted to determine MICs according to the Clinical and Laboratory Standards Institute (CLSI) guidelines. Firstly, 120 μL of different concentrations of oxacillin (0.031 - 64 μg/mL), vancomycin (0.125 - 64 μg/mL), meropenem (0.004 - 64 μg/mL) and levofloxacin (0.004 - 64 μg/mL) were mixed with 1.68 mL of agar medium, respectively. The quality control strains of bacteria including *Escherichia coli* (*E. coli*) (ATCC 25922), *Pseudomonas aeruginosa* (*P. aeruginosa*) (ATCC 27853) and *S. aureus* (ATCC 29213) were planted on the surface of agar plates in 3 duplicates. MICs were got after incubation for 18 h. Secondly, one milliliter of different concentrations of XBJ (0.1 - 100 μL/mL) in agar were prepared and then were mixed with 14 mL of agar medium. Eight common clinical bacterial strains including *E.coli* (ATCC 25922), *P. aeruginosa* (ATCC 27853), *S. aureus* (ATCC 29213), *K. pneumoniae* (ATCC 700603), carbapenem-resistant *Klebsiella pneumoniae* (CRKP) (HS11286), hypervirulent *Klebsiella pneumoniae* (hvKP) (GN-3), MRSA (ST239), *S. aureus* (ATCC 6538) were planted on the surface of agar in 3 duplicates. MICs were read after incubation for 18 h.

Cell proliferation assay

Cell viability of Raw264.7 in the presence of XBJ was measured using cell proliferation assay with cell counting kit-8 (CCK-8) according to the manufacturer's instructions (Genegen Biotech, Shanghai, China). Briefly, Raw264.7 cells were seeded in 96-well plates at a density of 2×10^5/mL and incubated at 37 °C for 24 h. The cell culture medium was subsequently replaced by medium containing different concentrations of XBJ (0, 3, 10, 30, 100 μL/mL). At the point of 24, 48 and 72 h, the optical density of each well was determined at 450 nm (with the reference wavelength of 650 nm) using a Synergy 2 Microplate Reader (Bio-Tek, Vermont, USA). At 2 h before detection, CCK reagent was added into the medium (10 μL/well) for

incubation. The amount of Raw264.7 was expressed as a percentage of untreated negative control.

RESULTS

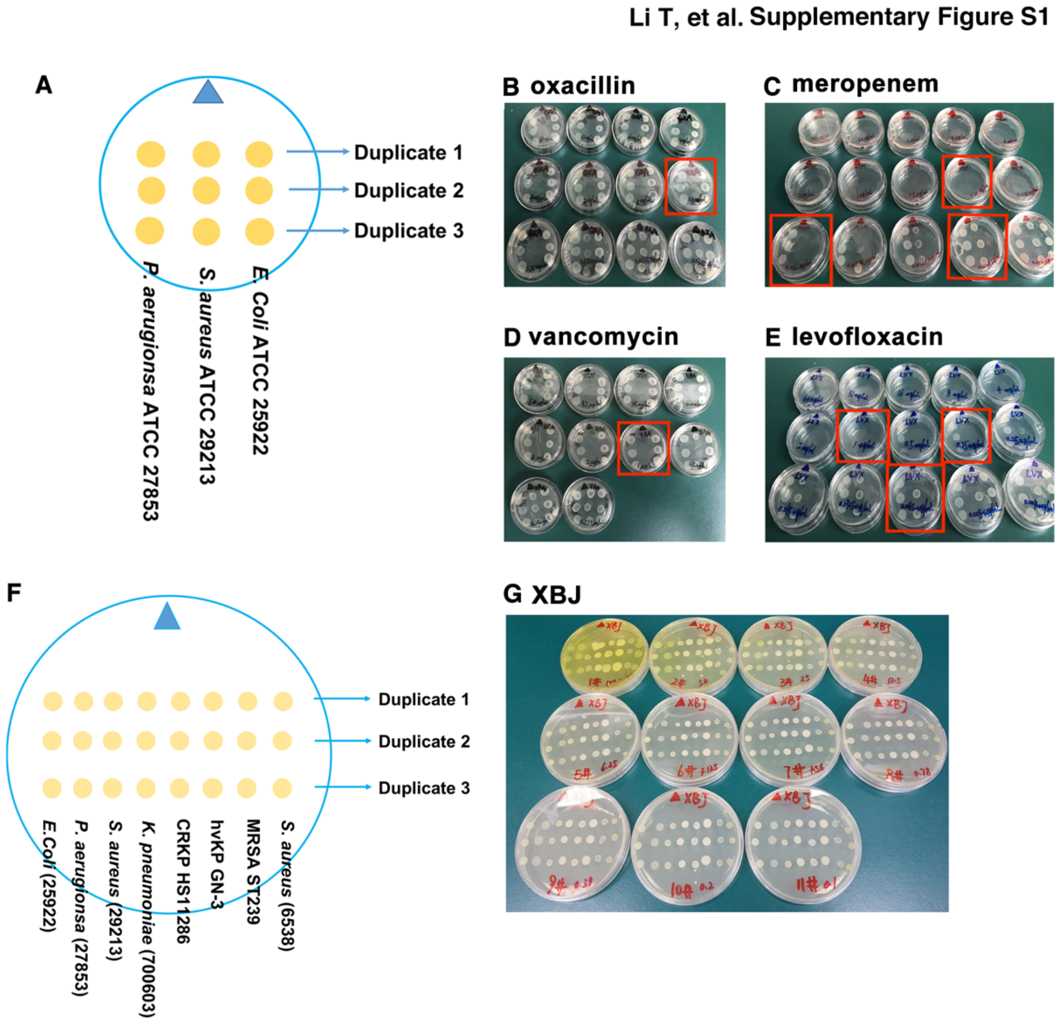

FIGURE S1 XBJ had no antibacterial effects within the examined concentrations (0.1 - 100 µL/mL). (**A, F**) The diagram of strains being planted on the surface of agar plates. (**B-E**) The red box indicted MICs data of quality control strains of bacteria to four examined antibiotics. (**G**) All examined clinical strains of bacteria grew on agar plates containing different concentrations of XBJ (0.1 - 100 µL/mL).

Li T, et al. Supplementary Figure S2

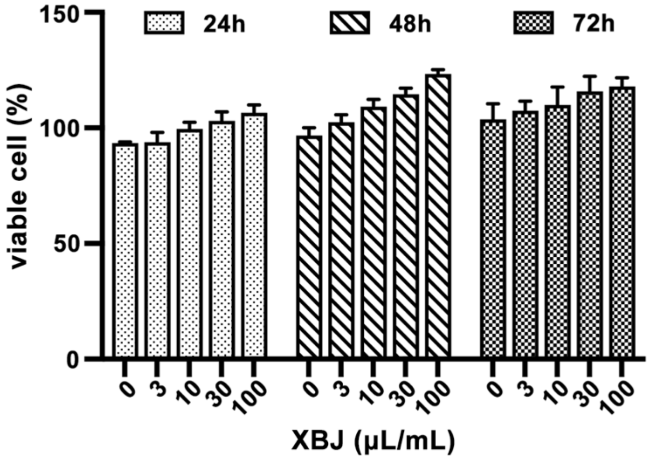

FIGURE S2 Effect of XBJ on the viability of Raw264.7. Cell proliferation assay was conducted to assess the cytotoxic effect of XBJ on Raw264.7. Data were shown as means ± SD (n=4).

Li T, et al. Supplementary Figure S3

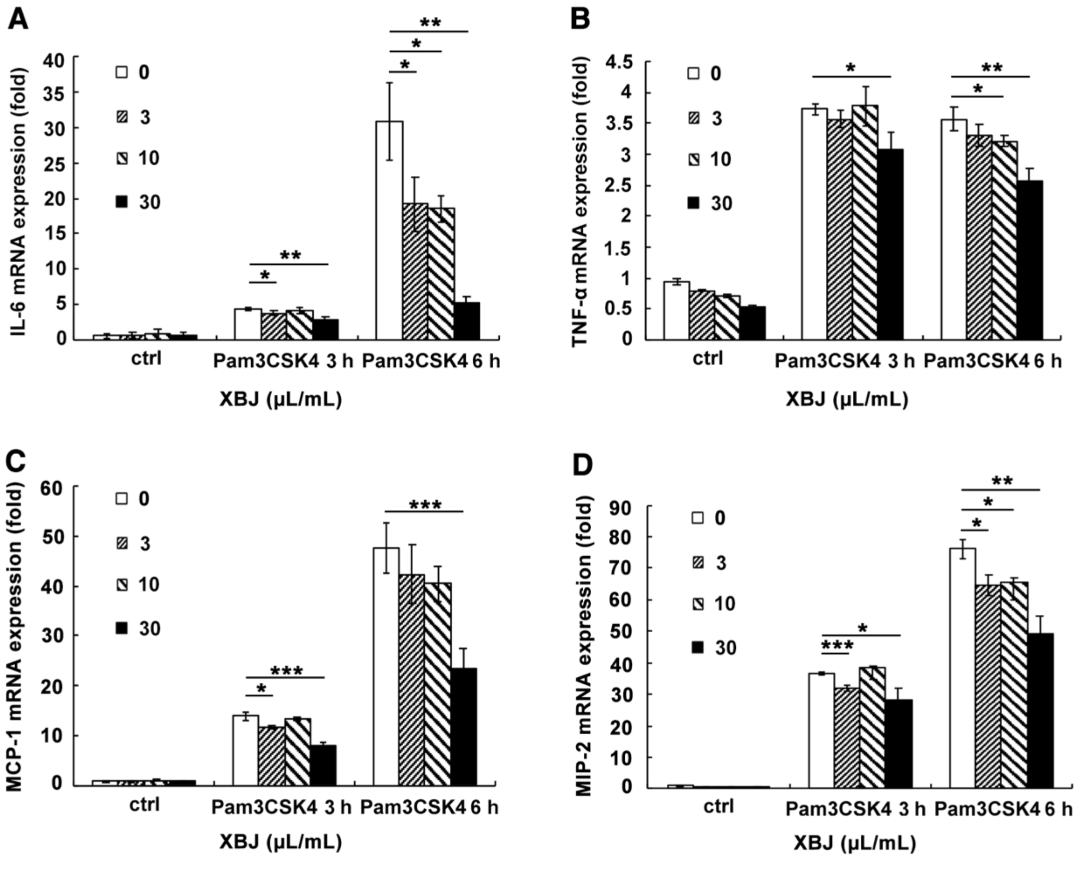

FIGURE S3 XBJ inhibited the production of Pam3CSK4-induced inflammatory mediators in Raw264.7. Raw264.7 cells were seeded (2×10^5 cells/well) in 24-well

plates overnight and stimulated by Pam3CSK4 (100 ng/mL) and different concentrations of XBJ for 3 or 6 h. IL-6 (**A**), TNF-α (**B**), MCP-1 (**C**) and MIP-2 (**D**) mRNA expression was examined by qRT-PCR. Data were shown as mean ± SD (n=3). *, **, ***Significantly different at $p < 0.05$, $p < 0.01$, and $p < 0.001$, respectively.

Li T, et al. Supplementary Figure S4

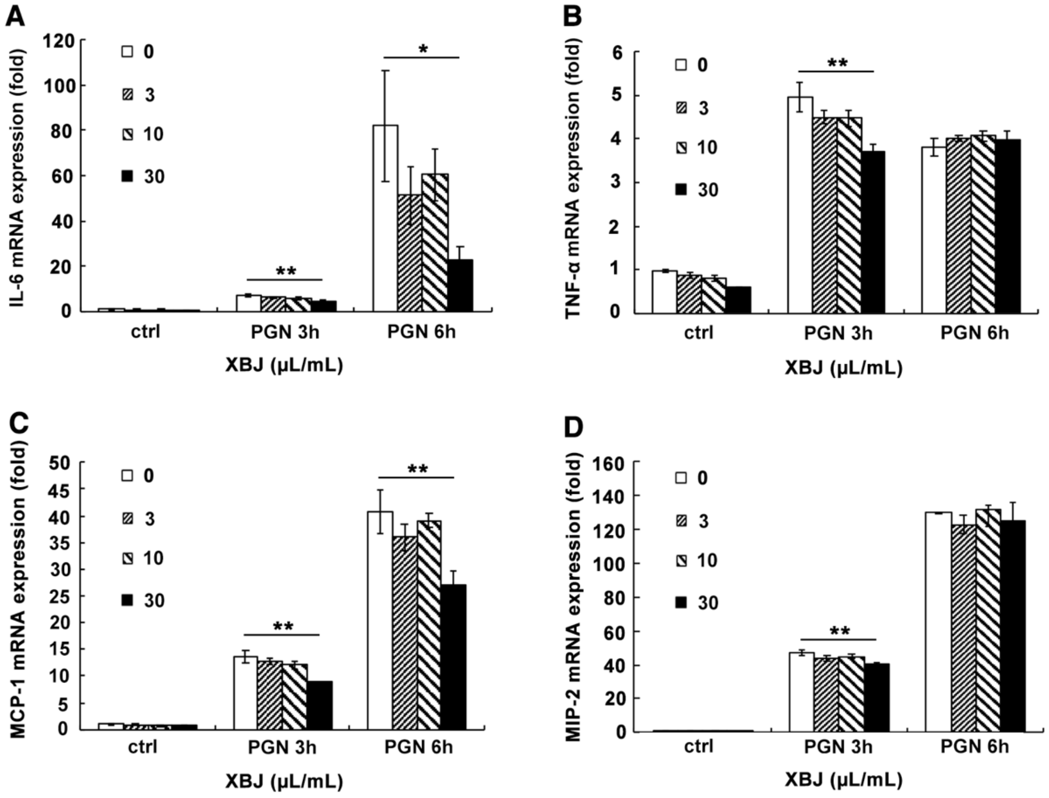

FIGURE S4 High dose of XBJ inhibited the production of PGN-induced inflammatory mediators in Raw264.7. Raw264.7 cells were seeded (2×10^5 cells/well) in 24-well plates overnight and stimulated by PGN (25 μg/mL) and different concentrations of XBJ for 3 or 6 h. IL-6 (**A**), TNF-α (**B**), MCP-1 (**C**) and MIP-2 (**D**) mRNA expression was examined by qRT-PCR. Data were shown as mean ± SD (n=3). *, **Significantly different at $p < 0.05$ and $p < 0.01$, respectively.

Table S1 MRM parameters of 9 compounds.

Compounds	Q1	Q3	t_R/min	DP(V)	CE(V)	CXP(V)
PAE	479.2	121	5.19	-100	-20	-15
HSYA	611.2	491.1	4.3	-60	-34	-34
ferulic acid	192.8	133.9	5.63	-31	-23	-10
tanshinone II A	295.2	277.1	10.81	58	27	21
salvianolic acid B	717.3	519.2	6.25	-100	-24	-22
benzoylpaeoniflorin	583.3	121.1	7.31	-54	-24	-15
alibiflorin	481.4	197.1	5.03	150	17	19
senkyunolide I	225.1	207.1	6.58	80	11	19
protocatechuic aldehyde	137	108	4.17	-100	-27	-15

Table S2 MICs of quality control strains of bacteria to four examined antibiotics.

	oxacillin	meropenem	vancomycin	levofloxacin
S. aureus	0.25<br>(0.12-0.5)	0.065<br>(0.03-0.12)	1<br>(0.5-2)	0.25<br>(0.06-0.5)
E. coli	invalid	0.008<br>(0.008-0.06)	invalid	0.016<br>(0.008-0.06)
P. aeruginosa	invalid	0.25<br>(0.25-1)	invalid	1<br>(0.5-4)

The values in brackets are concentration ranges recommended by the CLSI.
